# Access Barriers to Dental Treatment and Prevention for Turkish Migrants in Germany – A Qualitative Survey

**DOI:** 10.3389/fpubh.2022.862832

**Published:** 2022-05-26

**Authors:** Kristin Spinler, Christopher Kofahl, Erik Ungoreit, Guido Heydecke, Demet Dingoyan, Ghazal Aarabi

**Affiliations:** ^1^Department of Prosthetic Dentistry, Center for Dental and Oral Medicine, University Medical Center Hamburg-Eppendorf, Hamburg, Germany; ^2^Institute of Medical Sociology, Center for Psychosocial Medicine, University Medical Center Hamburg-Eppendorf, Hamburg, Germany; ^3^Department of Periodontics, Preventive and Restorative Dentistry, Center for Dental and Oral Medicine, University Medical Center Hamburg-Eppendorf, Hamburg, Germany

**Keywords:** migration, migrants, cultural sensitivity, oral health, dental prevention, oral health knowledge, dental treatment utilization, access barriers

## Abstract

**Introduction:**

The worldwide migration movement is growing and thereby challenging the health care systems of immigration countries like Germany to make health care equally accessible for all people. Due to their low oral health status and low uptake rates of dental treatment and prevention, migrants were detected as a vulnerable group. Data regarding dental care access barriers of this group is limited. Therefore, the following study established a deeper understanding of unknown access barriers.

**Methods:**

Nine expert interviews and one focus group interview were conducted semi-structured via interview guideline in the period of August until October 2018. The experts were persons with strong vocational interactions and experiences with the sector oral health care and migration. The focus group participants had a Turkish migration background.

**Results:**

The expert and focus group interviews revealed a variety of barriers that exist toward dental treatment and prevention for migrants. Language, perceived significance of oral health, oral health knowledge, health socialization and patient-dentist interaction were detected to be the main barriers with underlying subthemes and interactions. Furthermore, a predominantly not precaution-oriented dental service utilization of migrants was underlined by the interviewees. Additionally, ways to reach a higher cultural sensitivity in oral health care were stated.

**Conclusion:**

With respect for research, there is a need for the integration of migrant-specific items when collecting health data from people. With respect for policy, there is a need for more structural and individual attention for promoting equal access to oral health care and prevention measures for people with a migrant background.

## Introduction

In 2019, worldwide, 272 million people migrated to other countries, about half of them as migrant workers, to high- and upper middle-income countries (1). Further common reasons for migration are: war, displacement, or poverty (2). Germany is a country with a long migration history (2) leading to today's culturally mixed population of around 27% of persons with a “migration background”. In Germany's population statistics, “migration background” is used for immigrants and/or children of immigrants (3–6). In the following we refer to them in a short form as “migrants”. The most common countries of origin in Germany are Turkey (13.3%), Poland (10.8%), Russian Federation (6.6%), Kazakhstan (6%), Rumania (4.6%), Italy (4.2%) and Syria (3.9%) (7, 8). The migrant population is very heterogeneous with respect to their immigration motives, social, religious and cultural background, residence status and legal status of residence (3–5).

Compared to native Germans, migrants are facing specific health challenges and have special health care needs (9). Studies show significant differences in general health status, health behavior, and utilization of health care. For instance, worse self-rated health, higher rates of diabetes, higher risk of obesity and depressive disorders, and lower life expectancy were found in those vulnerable groups (8, 10–12). Next to socio-economic status (SES) and level of education (13), language barriers (14) have been identified as a main risk factor for both physical (13) and mental health (15).

These observations have been found to be true for oral health as well. Studies in different countries revealed a lower oral health status among migrants compared to born natives (16–21) even if controlled for SES and education in multivariate analyses (22). Thus, migration background has to be assessed as an independent risk factor for poor oral health. A nation-wide representative survey on health of children and adolescents (KiGGS) (23) as well as a study by Bissar et al. (24) show comparable results in children of immigrants. Further, children, whose both parents are immigrants, brush their teeth significantly more infrequent than those with at least one German born parent (25). Concerning oral health behavior and oral health care utilization, migrants are using oral health care services for preventive measures less often than the native population, and more often because of oral health problems and acute care needs (14, 18, 22, 26–28).

Despite this evidence, migrants are not sufficiently taken into account in representative oral health surveys (29).

To achieve a better understanding for oral health inequalities, how to strengthen the oral health of migrants at risk, and how to provide more culture sensitive and thus easier access to treatment and prevention, it is important to identify the barriers, which migrants are facing – specifically in their own view and based on their own experience. A few studies have investigated the access to dental care for different migrant groups. The hitherto most common barriers were language difficulties (30–34), low oral health knowledge and information (30, 33, 35), lack of transportation (30, 32, 33, 36), costs of dental treatment (31–33, 36, 37), and lack of insurance coverage (30, 37). Velez et al. additionally examined discrimination experiences, the type of immigration status, lack of social support and dissatisfaction with providers as well as long waiting times in a group of Mexican immigrants in the United States (37). A systematic review with focus on dental care of older migrants in developed countries showed inappropriate self-perception of dental care needs and cultural habits as barriers (32). Two other studies in New Zealand and the United States further revealed influencing aspects such as lacking of a regular dental practitioner (30), difficulties in making appointments, and a low priority given to oral health care (33).

These barriers may weigh differently in different countries and migrant groups due to their migration experience and cultural background as well as the specific health care system of the host country. To our knowledge, data regarding access barriers to dental treatment and prevention for migrants in Germany is poor, but needed (14, 26). Goal of the following study was to add further knowledge and deeper insights in experiences and opinions of migrants regarding oral health care. Because of the very high diversity and heterogeneity of the migrant population in Germany, we focused on the biggest migrant group, the residents of Turkish origin.

## Methods

### Study Design

Our study is based on an explorative qualitative study design (38). Between August and October 2018, nine expert interviews and one focus group with seven participants were conducted in Hamburg, Germany. Following a theoretical sampling approach (39), the experts for this study should have been persons who have strong occupational experiences with both oral health care and migration. The criteria for being an expert are based on the definition of Bogner et al. (40). Experts are described here as persons with a specific practical or experiential knowledge related to a clearly definable problem area. In addition, they are able to use their interpretations to structure the field of action for others in a meaningful and action-guiding way. The focus group participants were migrants from the largest group of immigrants in Germany (Turkey). Next to the experts with a professional background, we also consider the focus group participants as experts who have a specific expertise based on their own experience. Thus, all study participants should function as representatives of these specific fields from different perspectives.

### Recruitment

The experts (*n* = 9) were chosen by a gatekeeper strategy, which means that persons who have a good overview of and access to potential experts are requested to name and invite experts for an interview (41). In this case, the two project leaders of the MuMi project “Promotion of oral health and oral health literacy of people with migration backgrounds” identified and contacted six appropriate interview partners. In a snow ball procedure, three further experts were identified in the course of the first interviews and were contacted by the interviewers. The ethics approval for the expert interviews was given by the UKE ethics committee (Lokale Psychologische Ethikkommission am Zentrum für Psychosoziale Medizin) No: LPEK-0027.

To add further point of views, in particular the view of patients, method triangulation was applied by conducting an additional focus group in October 2018 with seven persons of Turkish origin who delivered personal insights in the themes 1. oral health care and 2. migration to “develop a comprehensive understanding of phenomena” (42). The focus on Turkish migration background has been chosen for two reasons: i) persons of Turkish origin form the largest ex-patriate community in Germany, and ii) this population group is known as having problems in health care usage (18) and health literacy (43).

Based on a theoretical sampling, focus group participants were recruited in different dental clinics by a dentist, who was part of the research team, and additionally via snow ball procedure by participants (39). Inclusion criteria were: Turkish migrants; 18 years or older; German or Turkish language; living in Germany. The participants were personally contacted and provided with written information about interview content and procedure. They gave an informed consent by signature and participated free of charge. The ethics approval for the focus group was given by the medical association Hamburg No.: PV5827.

### Data Collection

The interviews were conducted by telephone following a semi-structured interview guideline (Table 1). Initially, the experts were asked to talk freely about their professional experiences with and their impressions of the topic “migration and oral health”. In the following, they were asked to provide their knowledge of and experience with the utilization of dental care of migrants, their impressions of differences in oral health due to cultural influences and diversity, as well as cultural factors on the motivation of migrants concerning their oral health care. Concluding, they were asked about their ideas and suggestions for better oral health care and possible prevention measures for migrants. All interviews were conducted in German, audio-recorded and transcribed word by word.

**Table 1:** Semi-structured interview guideline for expert interviews.

	**Semi-structured interview guideline for expert interviews**
1	Can you please tell me, in which profession you are working and what your intersection and your experiences thereby are with the topics migration and oral health?
Oral health status:	
2	What do you know about the health status of persons with migration background? Do you know about differences and their reasons compared to non-migrants?
3	Which factors are most important for persons with migration background regarding their oral health? *(Look?, pain?, money?,…)*
4	Did you come across cultural characteristics and habits that can have an impact on oral health?
5	What do you know about the oral health literacy and oral health knowledge of our target group?
Oral health care utilization:
6	What are typical reasons and moments for persons with migration background to visit a dentist?
6.1	And furthermore, how is the participation in oral health prevention programs?
6.2	Can you see differences compared to non-migrants?
6.3	If yes, what are the differences and what could be the underlying reason for those?
7	Due to your knowledge, are persons with migration background facing special barriers towards the use of oral health care and prevention?
7.1	If yes, which are those barriers?
7.2	If no, what do you think is then the reason for differences in oral health status in contrast to non-migrants?
Motivation:	
8	How do you experience the motivation of the target group to look deeper into the topic of oral health and care about own oral heath behavior?
9	Do you see precise need and course for action to strengthen this motivation?
Prevention programs:
10	Do you think cultural characteristics of different groups should be considered in oral health prevention programs?
10.1	If yes, which should be considered and how?
10.2	If no, why do you think this is not necessary?

The focus group was semi-structured by a guideline that only slightly differed from the professional experts' one and was conducted by two researchers, a male dentist who is practicing in a dental clinic and a female health scientist in the field of migration and oral health. All participants and interviewers were fluent in German. After an introduction of topic and purpose of the focus group by the two interviewers and an introduction round by the participants, the group discussed several rounds of different questions (Table 2) around oral health care, cultural determined influences on self-care and access to dental health care etc.

**Table 2:** Semi-structured interview guideline for focus group interview.

	**Semi-structured interview guideline for focus group interview**
1	Starting with a wide and open question: Which meaning does health in general and oral health in particular have for you?
2	Do you think this is influenced by your past and the cultural influence of your families?
3	Where did you get your knowledge about oral health from in the past?
4	And which are your sources today if you have a question regarding oral health?
5	What are typical reasons for you and people in your surroundings to go to the dentist?
6	Which sorts of oral health prevention do you know and practice?
7	Is oral health an important topic in your communities? Do you talk about it?
8	So that you know both cultures, the Turkish and the German one, do you observe any differences by dealing with the topic oral health and in oral health behavior?
9	Do you experience any barriers that make it difficult for you or people of your community to go to the dentist or perform oral health prevention?
9.1	If yes, do you have ideas that could help to overcome these barriers?

The interviewers and participants developed a content mind map [knowledge mapping (44)] with key themes and statements during the group discussion. The focus group ended after 1 h and 50 min due to content saturation, agreed by all participants.

### Data Analysis

The qualitative analysis of the interview and focus group transcripts was performed on the basis of Creswell's content analysis (38) using the qualitative analysis software MAXQDA. During this process three levels of themes were developed: main, middle and sub (Table 3). Those themes were partly generated deductively, based on the prior knowledge of the researchers, and partly emerged inductively out of the content. After all themes were revised, all original transcripts were re-checked against them by two of the authors. Saturation was assessed as given, since the amount of overlapping information between the different study participants was significant. The knowledge mapping results were additionally included in the analysis and themes development. This hierarchy led to a theoretical model and conceptual framework as shown in the results section. All direct citations that are presented there were analogously translated into English.

**Table 3:** Theme system: main, middle and sub themes.

**Main themes**	**Middle themes**	**Sub themes**	**Named by:**		
			**Both**	**Expert**	**Focus**
Reasons for dental visits	Not precaution-oriented	Emergency	X		
		Pain	X		
		Bleeding		X	
Barriers	Language	Little knowledge of German	X		
		Misunderstandings		X	
		Illiteracy		X	
	Perceived significance	Little communication about oral health			X
		Low awareness for the importance of oral health	X		
		Socio-economic status/employment			X
	Knowledge	Deficits in knowledge about oral health		X	
		Little knowledge about the German oral health system		X	
		Difficulties in filtering right information (oral health literacy)	X		
		Fear of pain and costs	X		
		Language	X		
	Health socialisation	Different health care systems	X		
		Less group prevention programs in young age		X	
		Children are shaped by their parents	X		
		Concepts of health and illness	X		
		Look is more important than real health	X		
		Patient-Doctor interaction	X		
	Dentist interaction	Language	X		
		Little information about patients		X	
		Bigger time expenditure due to communication problems		X	
		Prejudices towards persons with migration background	X		
		Stigmatisation	X		
Cultural sensitivity in dentistry	Diverse pictures of food and persons			X	
	Simple language in descriptions		X		
	Pictures and videos complementary to descriptions			X	
	Multilingual dental practices			X	
	Awareness campaigns for the importance of oral health			X	
	Culturally sensitive dentists		X		

*
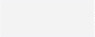
= deductive.*

*
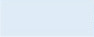
= inductive.*

## Results

### Sample Characteristics

All *expert interviewees* (*n* = 2 female, *n* = 7 male) had professional experiences in the fields of oral health and migration: two dental practitioners (dentist and dental prophylaxis assistant), two experts in dental migrant research, one expert in health sciences with focus on migration and health. The others worked in public organizations and services: one member of a public health service, one representative of the association of health insurance companies, and two representatives of the largest German dental boards (such as the Kassenzahnärztliche Bundesvereinigung Germany). Their experience in the field ranged from 3 to 25 years.

All *focus group participants* (*n* = 7 male, 28 to 60 years) had a Turkish migration background; two were born in Turkey, the others have at least one parent who was born in Turkey. All participants had collected experience with dental treatments and/or prevention.

### Themes

The interview participants reported a variety of barriers and challenges concerning dental treatment access and prevention measures for migrants. These were subsequently organized in the themes: language, perceived significance, knowledge, health socialization, and dentist interaction with patients. Next to barriers, the participants named different predominant reasons of migrants to visit a dentist (dental service utilization) as well as ideas how to shape dental care that is more culturally sensitive. The interview content has been structured in themes with their interactions and complemented by a theoretical framework that was developed on this basis (**Figure 2**).

#### Dental Service Utilization

The dental service utilization of migrants is consistently perceived as not precaution-oriented. Accordingly, many migrants seem to visit the dentist too late, e.g. in emergency situations, when they experience strong symptoms like pain, rather than earlier for preventive reasons.

“*What dentists were saying, was, for example, that the moment to which persons with migration background seek out for the dentist is different. This means, mostly when it's already dramatic, and the pain is strong; and they rather don't take advantage of prevention offers.” (Expert – dental migrant research)*

The experts perceive this behavior as a consequence grounded in barriers like a lack of oral health knowledge, unconsciousness of both meaning and aims of oral health prevention, misunderstandings due to language barriers, or fear of high treatment costs. These barriers are addressed in the following sections.

#### Barriers

The main theme “barriers” summarizes factors, situations, beliefs and views with the potential to hinder migrants to perform sufficient oral health care prevention, including visiting the dentist on a regular basis independent from symptoms.

##### Language

All interviewees see language as one of the key barriers. A lack of German language skills causes misunderstandings, lack of knowledge and information, misinterpretation of or missing out information, or difficulties in the patient-doctor communication.

“*Depending on the migration status, I would say that language barriers play a role. Of course, culture is more than language.” (Expert – migrant health research)*

One participant of the focus group described the possible consequences in a very emotional way:

“*The doctors don't even take the time for it, they think: ‘Oh, the guy doesn't understand me anyway. I don't give a shit, - next one. The main thing is that he comes next time.' I experienced that with my own parents.” (Focus group)*

Additionally, the experts emphasized that a considerable number of people, the majority being older migrants, are illiterate even in their mother tongue. This means that even written information in different languages would not be an option for these patients. Interpreters would be necessary. This problem was also discussed in the focus group. One participant suggested a structured professional solution for patients with insufficient German language skills:

“*Certain medical practices […] could specialize with specific appointments where mother-tongue treatment is provided.” (Focus group)*

Currently, however, professional interpreters are not paid by the statutory health insurance funds. If an interpreter is required, patients who do not speak German or another language that also the dentist or physician speaks have to organize an interpreter themselves, which in most cases are relatives or members of their migrant community.

##### Perceived Significance

Several participants of both groups clearly stated that in many other countries oral health would have a much lower value and significance than in Germany. They said that especially in Turkey, Afghanistan or Iran, for example, many people would simply not care about oral health and oral hygiene as it is not a high priority in these societies where the immigrants come from.

“*The perceived significance of oral hygiene measures, the regular dental care of persons with migration background is not comparable with the German population.” (Expert – German dental board)*

In both groups, a further common problem that was highlighted was little awareness of the importance of oral health care and its possible long-term consequences up to the impact on general health. Oral health would be a topic that is only rarely talked about with friends and family.

“*One has little time for things that are important. And it is not important to visit the dentist when one has no ailment.” (Focus group)*

The focus group participants claimed that there would be no time to give oral health a higher significance also because of the type of jobs many migrants are working in, usually not well paid (e.g. post man, craftsman, …). The base conditions and requirements of these jobs are not giving personal care in general, and oral health in particular, a high significance. For instance, some employees think they would not have the right to leave work for doctor appointments.

“*…time pressure, financial problems. Prevention moves in the background. Why should I visit the dentist if I don't have ailment? I have different problems. I need to be at work punctually. I cannot take the time to visit the dentist just to let him take a look over. I think that has less to do with migration than with general social problems in Germany.” (Focus group)*

In addition to the selected quotations presented here, there are many others, which - reading between the lines - suggest that there is considerable occupational pressure and/or financial distress, which negatively affects personal health self-care. With regard to the regular oral health check-ups offered up to twice a year in Germany, the focus group participants describe a strong dependence from their supervisors and their work conditions. Although being aware of these medical offers, they are hindered from taking advantage of them.

*(Person 4): “Society demands that you pay attention to preventive medical check-ups. So it's a contradiction in terms. It is not accepted at all by society in the case where it costs time. You can't go to your employer and say, ‘I need a bit of time tomorrow morning because I have to go to the dentist'. – ‘Do you have any ailments?'– ‘No.' – ‘Then you don't have to go to the dentist either'.” (Person 1): “Exactly.” (Person 4): “That's how it is with most employers, I suppose, normal employers where you go to work. And not the ones where you have a high position somewhere.” (Focus group)*

It seems obvious that perceived significance of oral health is interdependently associated with both social status and migration background. It further seems that the social environment does not support migrants (or blue-collar workers in general) to make use of preventive medical check-ups – in contrast to higher positioned white-collar staff. (By German law, preventive check-ups have to be arranged outside working hours, unless there is no possibility to get an appointment during free time.)

In this context, one focus group participant also described the significance of his own appearance as seen by others in Germany and how this affects him emotionally. This leads into a vicious circle undermining his self-confidence. He argues that appearance or looks would not be such a problem in Turkey.

“*One does not dare to smile. You have a worse appearance. You feel that if you can't open your mouth to laugh during a conversation because you are ashamed, for example, then you also have a worse self-confidence, I think, in this society here. And I think that in Turkey, in the same situation, it's not quite as bad as here.” (focus group)*

The combination of the described examples above could be summarized as a kind of societal discrimination of “lower class” persons, at least compared to “higher class” people who can rely on a higher tolerance and flexibility in combining their personal affairs with occupational demands.

##### Knowledge

Deficits in oral health knowledge, e.g. in the fields of healthy behavior, nutrition, or specific risk factors for oral health, were identified as a barrier toward dental treatment and prevention by both the professional experts and the focus group. Knowledge would help to understand the importance of oral health care is the main concept of most study participants. However, oral health knowledge would be associated with people's educational level.

“*(Among migrants) there was only little knowledge about oral health measures. With respect for the different backgrounds certainly understandable; but the problem of caries as a health threat – as it is socialized in Germany –, is not established – that one considers it as important to reduce this disease by the improvement of their own oral hygiene.” (Expert – German dental board)*

The study participants of both groups also named several unhealthy nutrition habits. Rituals for children were described, like putting honey on the pacifier to calm down to sleep or giving sweetened tea as main source of liquid. In some cultures, there are no intentions to restrict the sugar intake of children as it is still seen as an important source of energy although nowadays this would only be a relic of the past. However, not the sugar intake itself would be the main problem rather than the lack of subsequent tooth brushing.

“*The sweet tea after dinner, after the social gathering. This is not the big problem I think, but moreover it is the lack of preventive oral hygiene afterwards (before going to bed).” (Expert – dental practitioner)*

The consequence of lacking oral health knowledge was very vividly illustrated by an older participant in the focus group, who grew up in a rural area of Turkey, using his own history as an example:

“*For example, uh, I lost more than half of my teeth when I was 12 or 13, right? And the rest were all with holes. Uh, well, in my time there wasn't a single toothbrush in the village where I lived, we didn't know that. Absolutely not. And we only looked at our parents and saw, so to speak, our fingers wet, also salt, and rubbed with it, that was the only thing: brushing our teeth with fingers and salt.” (Focus group)*

Lack of knowledge about the German health care system and difficulties to filter the existing oral health information can potentially lead to insecurity and even fear. For example, fear of high dental treatment costs and fear of strong pain during dental treatment would hinder persons to visit the dentist. Bad experiences (own or parents') in other countries, where it is not mandatory or possible to use local anesthesia during tooth treatment or extraction, were seen to reinforce this fear. Especially the focus group participants discussed how the handling of fear regarding dentists is shaped by their cultural background including the takeover of their parents' painful experiences, which manifested in fear.

“*And he (the dentist) was walking around the whole day and when you had toothache he came, extracted, salt on it and that was it. There was no anesthesia, there was nothing, nothing. It (the tooth) was just completely extracted. And there it starts among us with the dental care and fear. When the dad tells this to the younger ones…” (Focus group)*

For the sake of completeness, however, it should be said that also in Germany the use of anesthetics in caries treatment (drilling) was not a matter of course three to four decades ago. Thus, most older Germans also had painful experiences with the dentist in their youth.

##### Health Socialization

Depending on the country of origin and the age of immigration, immigrants were socialized in different health care systems, which among other factors were named by the experts to influence oral health behavior and beliefs. For instance, out of pocket payment for treatments would reinforce the avoidance for dental check-ups, or they delay a necessary visit of the dentist until it is unavoidable.

“*Also, the question how the health care system is functioning in those countries. That means that the access to dental care is correlated with many barriers. One visits the dentist rather pain oriented, problem oriented, and not preventive to regular dental check-ups. The precaution orientation is not nearly on the same necessary level.” (Expert – German dental board)*

Additionally, persons who grew up in a different health care system may have not experienced early oral health prevention in groups as it is performed in nurseries, kindergartens and primary schools in Germany. It was mentioned by the experts that the concept of prevention as it is common in Germany is not understood by all persons who were socialized in a health care system in which prevention does not play a key role.

“*Prevention is something that is propagated in campaigns throughout Germany, but also something that needs to be asked for actively in some points. It is influenced by the organization of a society – is the focus on me or on the collective? Am I used to get things ordered from the top or do I have to ask for things actively? That is something that might also be different in different cultures.” (Expert – migrant health research)*

##### Dentist Interaction With Patients

The interaction between dentists and patients itself was in both groups seen as potentially obstructive, too. Not surprisingly, language was named as a crucial problem again. The focus group members emphasize the high relevance of doctor and patient understanding each other by speaking the same language (not necessarily German). This would be very important for full information exchange and trustful treatment. Dentists would need to obtain full information about the patient's symptoms and dental medical history to set up an effective and low-risk treatment plan. On the other hand, patients need to understand the entire treatment plan, potential costs, and reasons for the treatment to feel comfortable and confident.

“*…and they (persons with different cultural background) communicate their ailment different than, I would say, the German average citizen is doing it. Rather reserved or exuberant, depending on the entity staying in the background. This leads to the situation that the dentist sometimes does not know all relevant medical information.” (Expert – German dental board)*

Additionally, the patient-doctor interaction was described by the experts as shaped by culture. For instance, the way women and men interact would influence how free and comfortable a woman feels about being treated by a male doctor.

Both groups mentioned that some dentists seem to avoid the treatment of migrants. One named reason by the experts is that handling “those patients” would be too time consuming in their tightly tacked treatment rhythm. Existing prejudices in dentists' behavior and communication during the treatment toward migrants would make it nearly impossible for migrants to visit a dentist with a positive feeling.

“*Because she is wearing a headscarf and has no good command of the language, she made the prior experience that one (the dentist) was not talking with her, but exclusively with her accompanying person. Various patients mentioned that they made this experience.” (Expert – migrant health research)*

#### Recommendations for More Cultural Sensitivity in Dentistry

**Figure 1:**
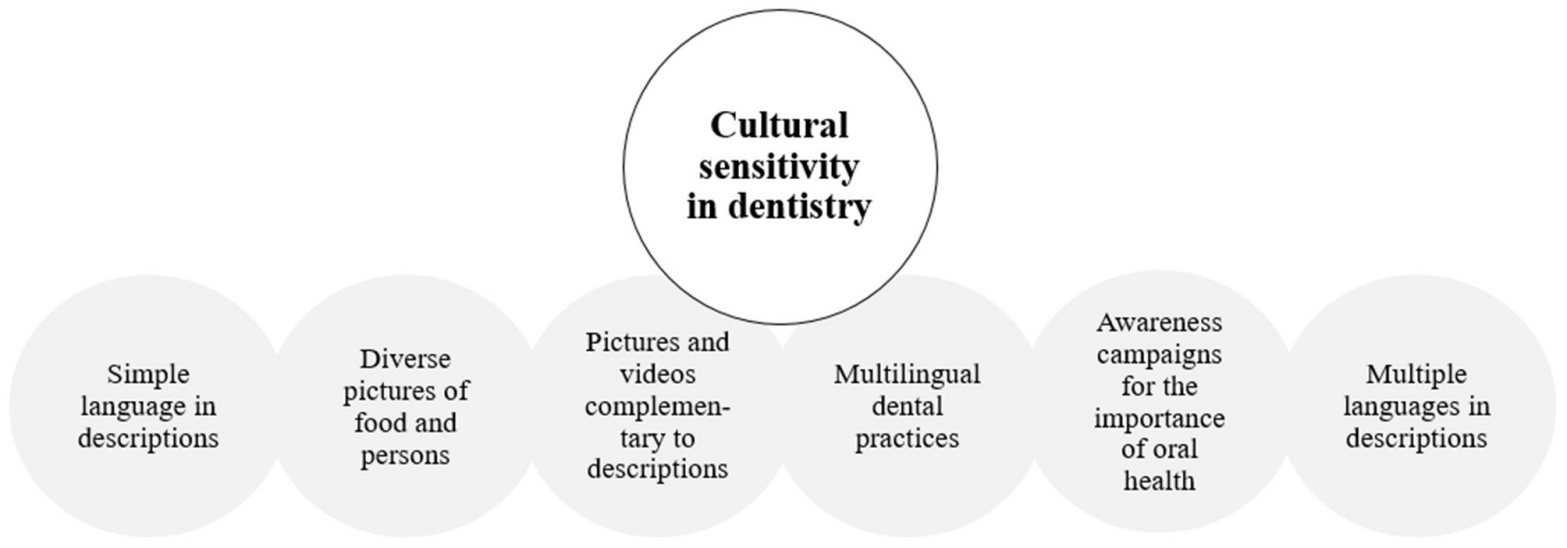
Cultural sensitivity in dentistry.

The interviewees gave a variety of recommendations to simplify the access to dental information, treatment and prevention (Figure 1). In order to minimize the language barrier, simple and multiple languages in written and spoken information should be used. Others focus on representation and inclusion of different cultures by picturing culturally diverse persons and food examples in dental information material and advertisement. Additionally, cultural sensitization of dental staff and sensitive awareness campaigns for the target group of migrants were recommended to improve the situation in dentistry.

**Figure 2:**
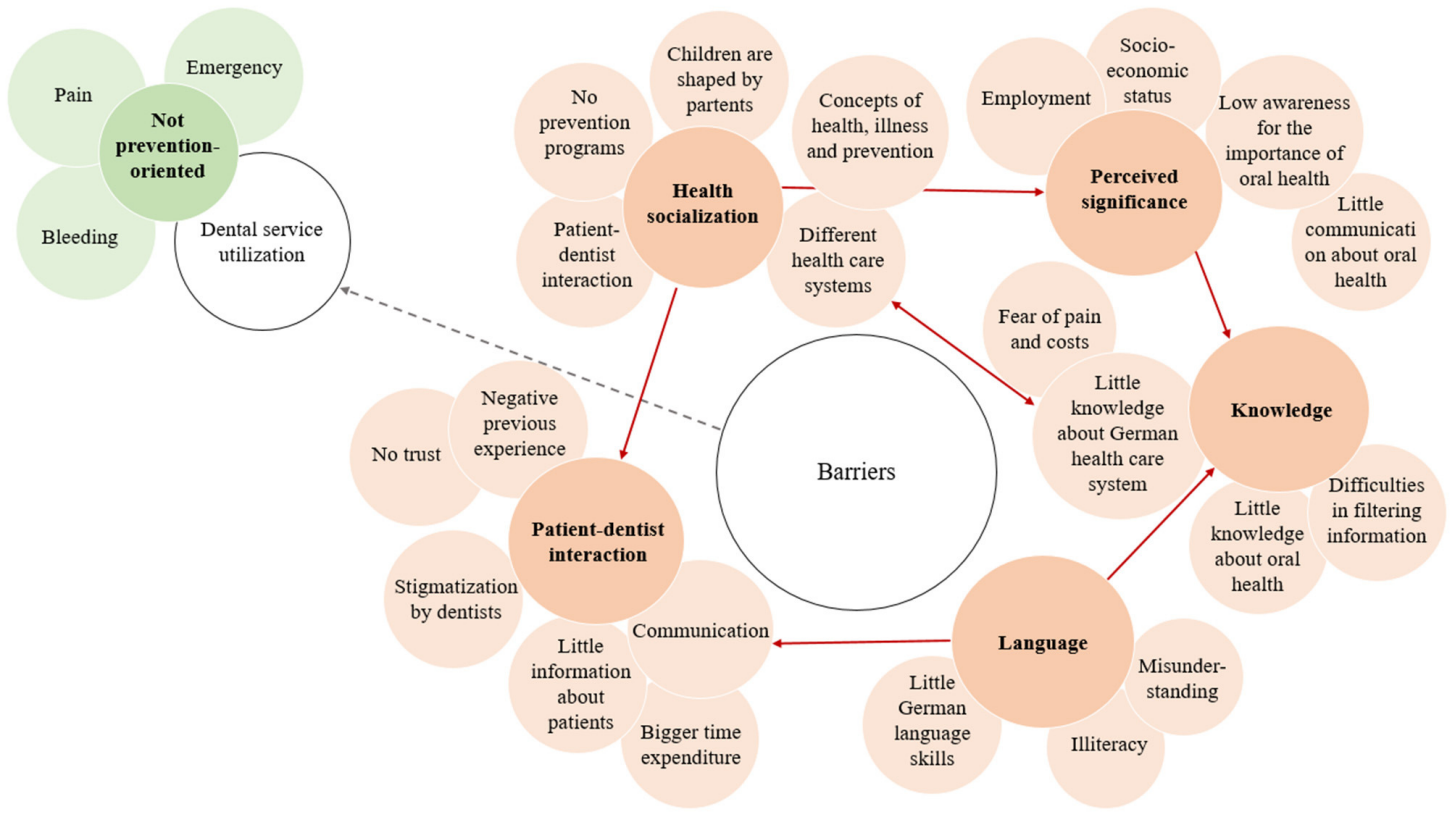
Barriers and reasons for dental visits for migrants in Germany.

The theoretical framework developed by the themes and their mentioned interactions is presented in Figure 2. The different themes overlap and influence each other. None of the identified barriers stands or exists on their own, as they interact and influence each other. Themes with white background present the identified main themes, the dark-colored ones symbolize sub-themes and the light-colored ones the related specific sub-sub-themes, e.g. knowledge (dark) and little knowledge about oral health (light). The red arrows show the direction of interaction, e.g. language barriers have an impact on persons' knowledge barrier.

## Discussion

This study revealed a variety of barriers existing toward dental treatment and prevention for migrants. Language, perceived significance of oral health, oral health knowledge, health socialization and patient-dentist interaction were detected to be the main barriers with underlying interacting subthemes. Furthermore, a predominantly not prevention-oriented dental service utilization of migrants was underlined by the participants. Additionally, ways toward a higher cultural sensitivity were identified, including the usage of simple and multiple languages in written and oral information, the inclusion of different cultures by depicting culturally diverse persons and food examples in dental information material and advertisement, cultural sensitization of dental staff, and sensitive awareness campaigns for the target group of persons with a migration background.

The presented results about the dental service utilization of migrants in Germany are in line with the available quantitative data. These, for instance, can be found in a study by Brzoska et al., which shows that migrants have a 36% reduced chance of utilizing regular dental checkups compared to persons without migration background (OR = 0.64). Language barriers and lack of knowledge and information on oral health topics seem to be some of the leading barriers for a higher dental service utilization of this group (14). So far, barriers toward dental treatment and prevention were not scientifically investigated in a qualitative study design in Germany.

The presented results extend the low number of existing quantitative research by some further themes like patient-doctor interaction and oral health socialization (14). The detected barriers have overlaps with findings in the field of prevention and general health as well (45).

The migration history of migrants might affect their today's oral health care utilization. As an example, the Turkish guest workers who emigrated in the 1960ies and 1970ies to Germany became one of the largest migrant populations in Germany although their staying for good was initially neither planned, nor desired, nor intended. This hindered the integration process and left both migrants and their descendants exposed to the stresses of the post-migration period (46). The in Germany born children of the Turkish guest workers learned German and went to school, but were of course still and mainly under the influence of their parents. Parents' oral health behavior is significantly associated with their children's oral health behavior and consequently their oral health itself (47). Therefore, many of them had only limited opportunities to learn and to live (oral) health prevention, namely to brush their teeth consistently and appropriately and to visit the (pediatric) dentist regularly.

The focus on Turkish migrants is often a look into the past – i.e. the look at their biography and the historical context, which also applies to the so-called second generation, i.e. their children. For oral health prevention, these children were born too early (in the 1970ies to 1980ies) to benefit from the very successful efforts of oral health prevention in nurseries, kindergartens and schools, which started intensively in the 1990ies and have been intensified until today. The third generation, the grandchildren, are in a much better situation with a significantly lower caries risk. Today, more than four out five children in Germany aged 12 never had caries; the social gradient, however, is still existent and dominant (48), admittedly on a much higher level.

The problems and challenges in oral health care, which Turkish immigrants had to meet in the past and still today, are not the same problems that immigrants and/or refugees have to face nowadays. Education and health care systems have changed and evolved around the world. We assume that the concept of “culture” as a specific risk factor for poor oral health is (not completely) melting away in the light of better education and increasing health literacy. In general, younger and better educated immigrants are likely to have fewer problems in finding and using health services; and they also will have a higher health awareness and a better oral care than the Turkish immigrants 50 years earlier. In summary, the focus should be on both i) older migrants with a low level of education and integration, and ii) independently from age, immigrants coming from underdeveloped countries.

As it was mentioned in the expert and the focus group interviews, health-related problems may be assessed as less important than other existing existential problems. Specifically, the participants of the focus group pointed out in detail that and how the personal job conditions and life situation are related to individual oral health care, but also how other people judge them based on the appearance of their teeth. This part of the discussion was emotionally charged and gave the impression that some of the participants felt hurt, up to losing their self-confidence, in the way they have been recognized and treated compared to other, better-off people.

Baumgarten et al. (49) found a strong association between dental appearance and discrimination in health care services. Interestingly, terms like discrimination, racism, racial prejudice, disadvantage or second class were not mentioned or used a single time in the focus group, although some of the examples concerning dentists' behavior could be interpreted as discrimination. However, the Turkish study participants consequently explained oral health risks and differences in oral health and oral health behavior with social and socio-economic differences, education, experiences in and from the past, language barriers etc., but not with possible personal characteristic related to their origin. In sum, all these factors in their combination may explain lack of knowledge, skepticism, avoidance of visiting a dentist, and perceived significance of oral health.

Overall, there is a lack of data concerning the oral health of migrants (29). At national level, a discussion about the development of strategies to improve the oral health of migrants has started only a few years ago (50). Institutions like the Robert-Koch Institute and the Institute of German Dentists, who regularly run nation-wide surveys, are considering higher migration sensitivity in their future studies (29, 48, 51, 52).

### Strengths and Limitations

The present study provides some of the few insights in subjective barriers toward dental service utilization and prevention that are experienced by Turkish migrants in Germany. A strength of this investigation is the triangulation approach that includes different point of views (experts and persons affected) on aspects that are rarely investigated in dentistry. It goes beyond the information that can be obtained when only experts talk about a target group. The developed theoretical framework and its graphical representation (Figure 2) can be used for training and education of dental practitioners and for further research. Certainly, it can and probably has to be further developed.

A limitation of the study is that the focus group consisted only of persons with a Turkish migration background. Therefore, the experiences and perspectives of other migrant groups could not be included. Furthermore, due to simplifications for the data collection, the interview questions did not differentiate between different countries of origin. It cannot be excluded that the semi-structured form might have set impulses that provoked generalized statements about migrants. Another limitation is that only two female participants could be recruited for the study. Reasons were a general difficulty to motivate persons for participation in the focus group due to limited time and transport capacities of participants; and the snowball procedure, which mostly resulted in male participants, has most likely motivated further male participants. The gender balance should be given more focus by offering separated interviews for men and women, performing the interview located close by participants e.g. in community buildings and focusing on balanced gender ratio while choosing the interviewees to include female experiences. When interpreting the results, it should be considered that these are based on subjective perceptions, which cannot be generalized to all persons with a migration background.

## Conclusion

The findings are an extension of existing quantitative studies as they give more insight behind the reasons for the differences between migrants and natives found in socio-epidemiological studies. With respect for research, there is a need for the integration of migrant-specific items when collecting health data from people. With respect for policy and public health, there is a need for more structural and individual attention for promoting equal access to oral health care and prevention measures for people with a migrant background. Further, migrant groups and countries of immigrants' origin are constantly changing. Consequently, health care research with focus on the volatile situation of migrants is a permanent process.

The developed framework shows that all analyzed factors are interacting and cannot be considered separately. In consequence, it seems to be necessary to empower migrants in all these different fields to reach higher oral health equality, which requires measures and support of professionals in the field of dental care, public health and education. This in turn needs intercultural competencies of the relevant stakeholders, which subsequently should be communicated and trained wherever necessary. The developed framework can be used as part of a concept or as a tool for training and education.

## Data Availability Statement

The datasets presented in this article are not readily available because they consist of qualitative interviews and focus groups that include sensitive information, which is not anonymized. Requests to access the datasets should be directed to GA, g.aarabi@uke.de.

## Ethics Statement

The studies involving human participants were reviewed and approved by University Clinic Hamburg-Eppendorf Ethics Committee (Lokale Psychologische Ethikkommission am Zentrum für Psychosoziale Medizin) No: LPEK-0027. The patients/participants provided their written informed consent to participate in this study.

## Author Contributions

CK and GA contributed to conception and design of the study. The semi-structured interview manual and the focus group guideline as well as the recruitment of experts for the expert interviews were performed by CK, GA, and KS. EU recruited the participants for the focus group and EU, KS, and GA conducted the focus group. Data analysis was performed by CK, DD, KS, and GA. KS wrote the first draft of the manuscript. CK, GA, DD, and KS wrote sections of the manuscript. All authors contributed to manuscript revision, read, and approved the submitted version.

## Funding

This publication was written within the framework of the project, MuMi: Promotion of oral health & oral health literacy of people with migration backgrounds. The MuMi-Project is funded by the Innovation Fund of the Joint Federal Committee (G-BA), Berlin, Germany (grant number 01VSF17051).

## Conflict of Interest

The authors declare that the research was conducted in the absence of any commercial or financial relationships that could be construed as a potential conflict of interest.

## Publisher's Note

All claims expressed in this article are solely those of the authors and do not necessarily represent those of their affiliated organizations, or those of the publisher, the editors and the reviewers. Any product that may be evaluated in this article, or claim that may be made by its manufacturer, is not guaranteed or endorsed by the publisher.
